# Identification of Filamin-A and -B as potential biomarkers for prostate cancer

**DOI:** 10.4155/fsoa-2016-0065

**Published:** 2016-12-22

**Authors:** Niven R Narain, Anne R Diers, Arleide Lee, Socheata Lao, Joyce Y Chan, Sally Schofield, Joe Andreazi, Rakibou Ouro-Djobo, Joaquin J Jimenez, Tracey Friss, Nikunj Tanna, Aditee Dalvi, Sihe Wang, Dustin Bunch, Yezhou Sun, Wenfang Wu, Khampaseuth Thapa, Stephane Gesta, Leonardo O Rodrigues, Viatcheslav R Akmaev, Vivek K Vishnudas, Rangaprasad Sarangarajan

**Affiliations:** 1 Berg, LLC, Framingham, MA, USA; 2 Department of Laboratory Medicine, Cleveland Clinic, Cleveland, OH, USA 44195; 3 Department of Chemistry, Cleveland State University, Cleveland, OH, USA 44115; 4 Department of Dermatology, University of Miami Miller School of Medicine, Miami, FL, USA 33136

**Keywords:** Bayesian network learning, biomarkers, FLNA, FLNB, KRT19, prostate cancer

## Abstract

**Aim:**

A novel strategy for prostate cancer (PrCa) biomarker discovery is described.

**Materials & methods:**

*In vitro* perturbation biology, proteomics and Bayesian causal analysis identified biomarkers that were validated in *in vitro* models and clinical specimens.

**Results:**

Filamin-B (FLNB) and Keratin-19 were identified as biomarkers. Filamin-A (FLNA) was found to be causally linked to FLNB. Characterization of the biomarkers in a panel of cells revealed differential mRNA expression and regulation. Moreover, FLNA and FLNB were detected in the conditioned media of cells. Last, in patients without PrCa, FLNA and FLNB blood levels were positively correlated, while in patients with adenocarcinoma the relationship is dysregulated.

**Conclusion:**

These data support the strategy and the potential use of the biomarkers for PrCa.

## Lay abstract

The goal of this study was to use a novel strategy that combines biological outputs with Bayesian network learning to identify potential biomarkers for prostate cancer (PrCa). This methodology identified two proteins, filamin B and keratin-19, as potential biomarkers for PrCa. The network map also identified a direct linkage between filamin B and filamin A, which is a protein that has previously been identified as playing a role in PrCa etiology. The identified proteins were then validated by examining their levels in a panel of PrCa cell lines and in human plasma samples.

**Figure 1.**
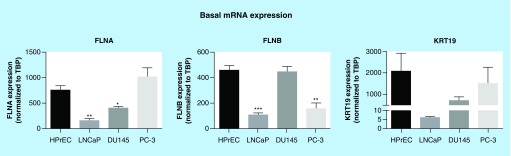
**Basal mRNA expression of biomarkers in prostate cancer cells.** Expression was assessed by quantitative RT-PCR and normalized to *TBP*. Data represent means + SEM, N = 3. * p < 0.05, ** p < 0.01 and *** p < 0.001 compared with HPrEC. HPrEC: Normal, human, primary prostate epithelial cells; FLNA: Filamin-A; FLNB: Filamin-B; KRT19: Keratin-19; RT-PCR: Real-time PCR; SEM: Standard error of the mean; *TBP*: TATA-binding protein.

**Figure 2.**
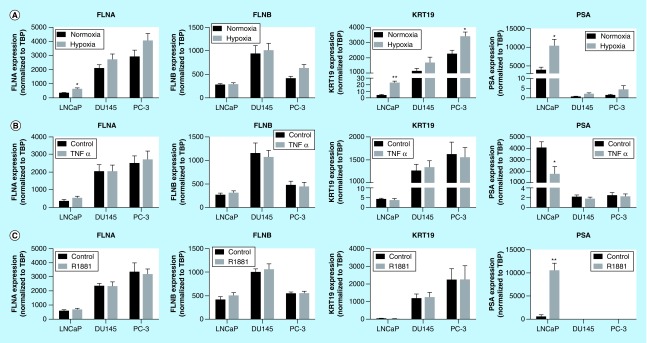
**Regulation of biomarkers by prostate cancer-relevant stimuli *in vitro.*** Cells were exposed to hypoxia (1% oxygen; **A**), TNFα (10 ng/ml; **B**) or R1881 (1 nM; **C**) for 24 h. Expression was assessed by quantitative RT-PCR and normalized to *TBP*. Data represent means ± SEM, N = 3. * p < 0.05 and ** p < 0.01 compared with normoxia or control. FLNA: Filamin-A; FLNB: Filamin-B; KRT19: Keratin-19; RT-PCR: Real-time PCR; SEM: Standard error of the mean; *TBP*: TATA-binding protein.

**Figure 3.**
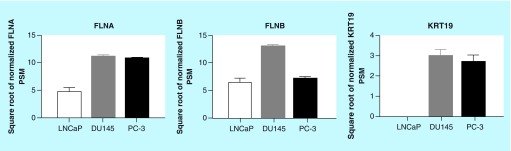
**Secretion of biomarkers from prostate cancer cells.** Conditioned media from cells were harvested and proteomic analysis was performed. Data represent means ± SEM, N = 3. FLNA: Filamin-A; FLNB: Filamin-B; KRT19: Keratin-19; PSM: Peptide spectral match; SEM: Standard error of the mean.

**Figure 4.**
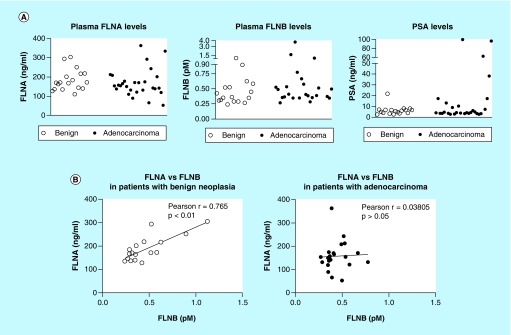
**Detection of filamin-A and -B in human plasma.** ELISA was used to detect FLNA and FLNB levels in blood samples from patients with suspected prostate cancer. **(A)** Scatterplot representation of FLNA, FLNB and PSA levels in patients. **(B)** Correlation analysis of FLNA and FLNB in patients with benign cases or adenocarcinoma. FLNA: Filamin-A; FLNB: Filamin-B; PSA: Prostate-specific antigen.

The prostate-specific antigen (PSA)-based blood test is used as a first-line assay for detecting prostate cancer (PrCa) [1,2]. However, due to its lack of accuracy it has led to over diagnosis and unnecessary aggressive interventions in patients with indolent disease. Thus, the US Prevention Services Task Force issued a recommendation against the use of PSA-based screening in 2012 [3]. Thus, there remains a critical unmet clinical need for identification of novel biomarkers to improve detection of PrCa.

Novel biomarkers that are currently in development for PrCa include the *TMPRSS2-ERG* fusion, *PCA3* and *AMACR*, each of which have been described for use alone or use in combination to improve the diagnosis of PrCa [4]. However, a major disadvantage of these biomarkers is the need for core biopsy specimens for assessment [4]. Thus, while these tests alone or in combination with PSA are clinically useful [4], discovery and development of biomarkers that can be detected in biofluids or any other easily available matrix would provide an advantage over existing markers in development.

Proteomic profiling of the secretome of cancer cells is a promising strategy that is employed to identify novel biomarkers for various cancers including PrCa. The ‘secretome’ is a term that was first presented by Tjalsma *et al.* based upon their findings from genome-wide screening of putative proteins that are secreted by *Bacillus subtilis* [5]. As secreted proteins may theoretically enter bodily fluids, their potential ability to be detected using noninvasive methods makes them a highly attractive target for biomarker discovery. Therefore, in the present report a novel data-driven systems biology-based approach was used to identify potential biomarkers for PrCa from interrogation of the secretome of PrCa cells (see Supplementary Figure 1 for schematic).

## Materials & methods

### Materials

All chemicals were obtained from Sigma-Aldrich and of analytical grade unless otherwise noted (MO, USA). Methyltrienolone (R1881) was obtained from PerkinElmer (MA, USA).

### Cell culture

Normal, human, primary prostate epithelial cells (HPrEC) (Lifeline Cell Technology, MD, USA) were maintained in ProstaLife media containing 6 mM L-glutamine, 0.4% bovine pituitary extract, 1 µM epinephrine, 0.5 ng/ml TGFα, 100 ng/ml hydrocortisone hemisuccinate, 5 µg/ml insulin and 5 µg/ml apo-transferrin. LNCaP and PC-3 PrCa cell lines (Sigma-Aldrich) were maintained in RPMI 1640 and high glucose DMEM, respectively. DU145 PrCa cells were maintained in Eagle's Minimum Essential Medium (EMEM). Base media were supplemented with 10% fetal bovine serum and 1× Pen/Strep/AmphoB. All cell lines were maintained in a humidified environment at 37°C under 5% CO_2_. All cells lines were authenticated using short tandem repeat DNA profiling (Genetica DNA Laboratories, Inc., NC, USA). For treatment with R1881, cells were incubated in charcoal-stripped fetal bovine serum-containing media for 24 h prior to exposure to R1881.

### Human plasma samples

Residual lithium-heparin plasma was collected from patients with suspected PrCa (see Table 1 for patient demographics). The criteria for inclusion were: elevated PSA results (≥2.6 ng/ml), age 45–70 years and ≥700 µl of sample. Samples were excluded if patients had any cancer diagnosis prior to collection. Biopsy information was accessed 6–8 weeks after sample collection. The diagnosis of benign, prostatic intraepithelial neoplasia or adenocarcinoma was noted. A Gleason score was recorded for patients with prostate adenocarcinoma. All procedures were performed in accordance with the ethical standards of the responsible committee on human experimentation (institutional and national) and with the Helsinki Declaration of 1975, as revised in 2000 and 2008.

### Identification of potential biomarkers using a data-driven approach

Cells were cultured in low (5 mM) or high (22 mM) glucose, with and without lactic acid (12.5 mM), and at normoxia (∼21% oxygen) or hypoxia (2% oxygen) (see Supplementary Figure 1 for schematic). Conditioned media were harvested at 24 and 48 h for proteomics analysis [6], and used as inputs for the Bayesian network inference model.

### Proteomic analysis of conditioned media

After cells were treated with various stimuli, 15 ml of conditioned media were collected and frozen at -20°C until use. Samples were thawed, then concentrated using a 30kDa MW cutoff filter (Amicon UFC903008, EMD Millipore, MA, USA) prior to centrifugation for 18 min at 4000 × *g* at 4°C. Protein concentration was determined using the Bradford assay. Up to 50 µg of protein was prepped using the filter-aided sample preparation method (Expedeon, CA, USA) and processed for proteomic analysis as previously described [6]. Briefly, samples were reduced by addition of 200 µl of 8 M urea and 10 mM DTT (Sigma-Aldrich), vortexed for 30 min at room tempreture (RT), transferred to filter-aided sample preparation spin filters and spun down for 10 min at 14,000 × *g*. This process was repeated for an additional time. Sample alkylation was performed by adding 10 µl of resuspended iodoacetamide and incubation at RT for 20 min. Samples were centrifuged and washed twice with 100 µl of 8 M urea and once with 100 µl of 50 mM ammonium bicarbonate. For digestion, 2 µg of trypsin (Sigma-Aldrich) was added to each sample. Samples were incubated at 37°C overnight with gentle linear shaking then eluted the next day with 40 µl of ammonium bicarbonate prior to centrifugation followed by elution with 110 µl of optima water. Samples were then dried down in a speed vacuum for 1.5 h and desalted using Pierce C18 desalting spin columns (Thermo Fisher Scientific, MA, USA). The desalted samples were dried down and resuspended in 20 mM ammonium formate.

Approximately 5 µg of sample was injected and run on a Waters 2D NanoAquity HPLC system coupled with a Thermo Q Exactive Plus Mass spectrometer (Thermo Fisher Scientific). The LC ran five fractions with 90 min gradients while the mass spectrometer (MS) collected full MS and MS/MS (Top 20) data over 500–1800 m/z. The acquired MS/MS spectra were analyzed with Proteome Discover 1.4 (Thermo Fisher Scientific) using a SequestHT search engine and a Human fasta database. Search parameters included 20 ppm for MS tolerance, 0.02 Da for MS^2^ tolerance and full trypsin digestion, which allows for up to four missed cleavages. Carbamidomethylation (C) was set as the fixed modification. Oxidation (M) and deamidation (NQ) were set as dynamic modifications. Peptides and protein identifications were filtered to allow a 99% confidence level of protein identification (1% false discovery rate).

### Bayesian network inference

Bayesian networking is a mathematical methodology developed for characterization of a multivariate system of random variables. The Bayesian networks approach is a framework where each particular factorization and the choice of parameters are a distinct probabilistic model of the process that created the observed experimental data [7]. A model ensemble of networks was built by using a Bayesian network learning and simulation software that combined the shotgun isobaric tag for relative and absolute quantitation (iTRAQ) proteomics data and the functional outputs [8]. The functional outputs include results of low and high glucose conditions, presence of lactic acid and oxygenation status [8]. Individual node *in silico* knockdown simulations were analyzed to refine the graph model. The expression level of each protein was reduced one at a time and the posterior distributions of all downstream nodes were statistically compared with their baseline distributions by calculating the c-statistic and the fold difference between the posterior and baseline distribution means.

The sub-networks linked to the functional variables were analyzed by determining their Burt’s constraint metric [9]. This measure calculates the extent to which nodes are connecting to unconnected modules and the relationship redundancy within each of these modules. Nodes with lower Burt’s constraint score are more likely to have a stronger effect on the network structure once it is bridged between nonredundant modules. The inferred molecular interaction networks represent localized casual pathways that may drive cytotoxicity so that the nodes with the lowest Burt’s constraint metric are potential biomarkers.

### Quantitative real-time PCR

Total RNA was extracted using a Qiagen RNAeasy kit per manufacturer’s protocol (Qiagen, MD, USA). First-strand cDNA synthesis was performed using 1 μg of RNA using the Invitrogen high-capacity cDNA reverse transcription kit (Thermo Fisher Scientific). Quantitative real-time PCR was subsequently carried out using the Bio-Rad CFX384 Real Time System (Bio-Rad, CA, USA). PCR primers for specified genes were used with iQ SYBR Green Supermix (Bio-Rad). TATA-binding protein primers were used with TaqMan Universal PCR Master Mix (Invitrogen) and served as a house-keeping control gene. All results were normalized to TATA-binding protein using the ΔCt method.

### Detection of PSA in human serum samples by electrochemiluminescence immunoassay

Total PSA testing was performed using the Elecsys total PSA immunoassay reagent (Roche Diagnostics, IN, USA), which measures total (free + complexed) PSA, on a Roche Cobas E602 instrument (Roche Diagnostics). The assay was performed per manufacturer’s recommendations. Briefly, 20 µl of sample was incubated with biotinylated monoclonal PSA-specific antibody and a monoclonal PSA-specific antibody labeled with ruthenium complex Tris(2,2′-bipyridyl)ruthenium(II)-complex (Ru(bpy)), which react to form a sandwich complex. Streptavidin-coated microparticles were then added to form a complex with biotinylated-labeled antibody. The reaction mixture was then aspirated into the measuring cell where the microparticles were then magnetically captured onto the surface of the electrode. Any unbound substance was subsequently removed with ProCell/ProCell M. A voltage was then applied to the electrode, which induced chemiluminescent emission that was measured by a photomultiplier. Results were then determined via a calibration curve generated by the instrument using a two-point calibration and a master curve provided by the reagent barcode.

### Detection of filamin-B in human serum samples by ELISA

Microtiter polystyrene 96-well plates (R&D Systems, MN, USA) were coated with 100 µl per well of 14 µg/ml coating antibody solution made with Antibody 5 (3F10 Antibody Group) diluted in phosphate buffered saline (PBS) (R&D Systems). Specifically, the plates were incubated overnight, washed with wash buffer (0.05% Tween-20 in PBS, pH 7.2–7.4) and blocked with 1% bovine serum albumin (BSA) in PBS, pH 7.2–7.4. During the assay, 50 µl of assay diluent was added to all wells followed by 100 µl of standard, control and sample per well. The standard was full-length recombinant human protein (Genscript, NJ, USA) in the range of 0.156–10 pM. The plate was securely covered with a plate sealer and incubated for 2 h at RT on an orbital shaker set at 180 ± 20 rpm. The plates were washed with 400 µl of wash buffer for a total of four times. Plates were then incubated with 200 µl of detection antibody (biotinylated filamin-B [FLNB]-specific monoclonal antibody, 0.1 µg/ml; R&D Systems) and incubated for 2 h at RT on an orbital shaker set as specified above and followed by washes. Samples were then incubated in 200 µl of Streptavidin-horse radish peroxidase (HRP) for 30 min, then washed prior to addition of 200 µl of substrate solution (a 1:1 mixture of H_2_O_2_ and tetramethylbenzidine) followed by incubation for 30 min at RT in the dark. The reaction was terminated with 50 µl of 2N sulfuric acid (R&D systems) and the plate was read at 450−540 nM using a BioTek plate reader (BioTek, VT, USA) within 30 min. A log–log curve fit was used to calculate sample concentrations.

### Detection of filamin-A in human serum samples by ELISA

Microtiter polystyrene 96-well plates (R&D Systems) were coated with 250 µl per well of 6 µg/ml coating antibody solution made with Antibody 4 (Berg ID 112597, Antibody Group) diluted in PBS. For the coating process, the plates were incubated overnight, washed with wash buffer (0.05% Tween 20 in PBS, pH 7.2–7.4) and blocked with 1% BSA in PBS, pH 7.2–7.4. During the assay, 100 µl of assay diluent was added to all wells followed by 50 µl of standard, control and sample per well. The standard was full-length recombinant human protein (Genscript, Berg ID 112054) in the range of 3.125–200 ng/ml. The plate was sealed and incubated for 2 h at RT on an orbital shaker set at 150 rpm. The plates were washed with 400 µl of wash buffer for a total of four times using a plate washer. Plates were then incubated with 200 µl of detection antibody (biotinylated filamin-A [FLNA]-specific monoclonal antibody, 15 ng/ml) for 2 h at RT on an orbital shaker set as specified above followed by washing the plate. Plates were then incubated with 200 µl of Streptavidin-­HRP for 30 min, washed and then incubated in 200 µl of substrate solution (1:1 mixture H_2_O_2_ and tetramethylbenzidine) for 30 min at RT, protected from light. The reaction was terminated with 50 µl of 2N H_2_SO_4_ and the plate was read within 30 min using a microplate reader at 450 nm with a 540 nm correction. A log–log curve fit was used to calculate sample concentrations.

### Statistical analysis

Proteomics data were processed by parsing the number of peptide spectral matches (PSMs) of each peptide from a spreadsheet generated by the instrument. Only PSM numbers whose q-values were <0.01 were used. Peptides from nonhuman proteins were removed. PSM numbers for same peptide sequences with different modifications were combined. Total PSM number of each sample was scaled to the median of total PSM number of all samples. χ^2^ test was used for differential analysis on each peptide across conditions. Since each condition has the same number of replicates, sum of scaled PSM numbers for each group was used in a χ^2^ test. p-value was corrected to false discovery rate for multiple tests.

All other data were analyzed using GraphPad Prism (CA, USA). For comparison between two groups Student’s t-test were performed. For comparisons with three or more groups one-way analysis of variance (ANOVA) followed by Dunnet’s posthoc analysis for multiple comparisons was performed. A p-value of 0.05 was deemed significant. Correlations were calculated using Pearson’s r.

## Results

### Selection of candidate biomarkers

In multiple types of cancers, metabolic and redox imbalances play a critical role in regulating cell signaling events, which in turn activates survival pathways, disrupts cell-death signaling and increases cell proliferation, and thereby promote a malignant phenotype (reviewed in [10,11]). A combined approach encompassing *in vitro* perturbation, high-throughput proteomics of conditioned media and *in silico* sub-delineation of causal networks and ranking of molecules using Burt’s constraint score was used to identify potential causal molecules of PrCa. FLNB and Keratin-19 (KRT19) were identified as very important nodes that were causally influenced by culture conditions of hypoxia and lactic acid in the metastatic prostate cancer cell line, PC-3 (Table 2). The Burt’s constraint score metric of FLNB and KRT19 are ranked at the top of the list (Table 2 & Supplementary Table 2). Of interest, FLNB showed a causal link to another filamin protein, FLNA (Supplementary Figure 2). Although FLNA was ranked at 52 (Table 2 & Supplementary Table 2), given its causal link to FLNB and the literature supporting its role in PrCa [12–14], FLNA was further studied to biologically validate the *in silico* findings and to examine the relationship between FLNA and FLNB.

### Characterization of mRNA expression of biomarkers *in vitro*

The identification of the molecules described above was derived from conditioned media of PC-3 cells (Supplementary Figure 1). Therefore, additional PrCa cell lines, LnCAP (androgen sensitive) and DU145 (androgen insensitive), were examined. Notably, the inclusion of an additional androgen-insensitive cell line that is less aggressive than PC-3 allowed us to compare across cell lines the basal mRNA expression and/or secretion abilities of the potential biomarkers. The basal mRNA expression levels of *FLNA*, *FLNB* and *KRT19* were characterized and compared with HPrEC (Figure 1). In LnCAP cells, *FLNA* and *FLNB* mRNA levels were significantly less than HPrEC. In DU145 cells *FLNB* mRNA levels were comparable to levels in HPrEC cell lines, while *FLNA* mRNA levels were significantly decreased compared with HPrEC cell lines. In PC-3 cells, mRNA levels of *FLNB* were lower than in HPrEC, but there was no difference in *FLNA* mRNA levels between PC-3 and HPrEC. *KRT19* mRNA levels were not significantly different across cell lines. These data demonstrate variation in mRNA expression of *FLNA*, *FLNB* and *KRT19* across primary and PrCa cell lines. Moreover, these data suggest that differences in mRNA expression of the biomarkers across cell lines do not appear to be related to aggressiveness or androgen dependency.

### Transcriptional regulation of biomarkers by prostate cancer-relevant stimuli *in vitro*

A number of microenvironmental cues are known to contribute toward PrCa etiology and progression including hypoxia, inflammatory stimuli and androgens [15,16]. In response to hypoxia a modest increase in *FLNA* mRNA expression (1.85-fold) was observed in LnCAP cells, but with no significant effect in DU145 and PC-3 cells (Figure 2A). *FLNB* expression was unaltered by hypoxia, while *KRT19* mRNA was induced by hypoxia in LnCAP and PC-3 cells. Consistent with previous reports [17], *PSA* expression was markedly increased in response to hypoxia only in LnCAP cells. Expression of select hypoxia-responsive genes (*GLUT-1*, *LDHA* and *Eno1*) was used as a positive control to confirm increased mRNA expression of these genes across cell lines and thus activation of canonical hypoxic responses in all cell types (Supplementary Figure 3A).

TNFα is an inflammatory stimulus known to drive PrCa development [18]. The expression levels of *FLNA*, *FLNB*, and *KRT19* were not regulated by TNFα exposure in any cell type (Figure 2B). In contrast, *PSA* expression was significantly decreased by TNFα exposure in LnCAP cells, consistent with previous reports that androgen receptor (AR) signaling is suppressed by inflammation [19]. Similarly, when LnCAP, DU145 and PC-3 cells were treated with the R1881, *FLNA*, *FLNB* and *KRT19* were not significantly affected, while *PSA* expression was significantly altered in LnCAP cells (19-fold increase; Figure 2C), which is expected for an AR target gene [20]. Notably, the treatments of androgen-insensitive cells with androgen stimuli were included as negative controls to confirm the lack of ability to induce mRNA expression of *PSA*. In these cell lines, none of the potential biomarkers were induced by R1881, as expected for androgen-insensitive cells. The expressions of inflammatory/NFκβ (*MCP-1*, *IL-6* and *COX-2*) and androgen- (*TMPRSS2*) responsive genes were generally upregulated across cell lines and included to confirm activation of canonical inflammatory- and androgen-responses after TNFα and R1881 exposure, respectively (Supplementary Figure 3). Together, these data demonstrate that the regulation of *FLNA*, *FLNB* and *KRT19* by PrCa-relevant stimuli is unlike *PSA*, suggesting their transcriptional regulation occurs through pathways independent of *PSA*.

### Secretion of biomarkers *in vitro*

The initial identification of FLNA, FLNB and KRT19 as causal markers of PrCa was derived from the secretome of PC-3 cells. Therefore, whether the biomarkers could be detected in conditioned media of LnCAP and DU145 cell lines was examined (Figure 3). FLNA and FLNB were detected in conditioned media from all cell types. KRT19 was detected in conditioned media from DU145 and PC-3 cells, but not in LnCAP cells. Together, these data indicate that KRT19 can only be detected in the secretome of DU145 and PC-3 PrCa cell lines, while FLNA and FLNB can be detected from the secretome of all PrCa cell lines examined.

### Assessment of plasma FLNA & FLNB levels

Given the ability to detect FLNA and FLNB in the secretome of all PrCa cell lines (Figure 3) and the *in silico* findings of a causal relationship between FLNA and FLNB (Supplementary Figure 2), FLNA and FLNB levels were assessed in human plasma samples from patients with suspected PrCa to determine proof-of-concept for detection in blood.

Patient demographics are shown in Table 1. There were no significant differences in age, PSA and mean number of biopsies between patients diagnosed with benign cases compared with those with adenocarcinoma. Notably, both FLNA and FLNB proteins were detected in plasma samples (Figure 4). However, there were no significant differences between groups in FLNA (mean ± SEM = 182.5 ± 15.22 vs 168 ± 13.39 for patients with benign vs adenocarcinoma, respectively) and FLNB (0.444 ± 0.06 vs 0.663 ± 0.15 for patients with benign vs adenocarcinoma, respectively) (Figure 4A). As level of PSA is the current method utilized in the clinic for initial assessment of patients with suspected PrCa, PSA levels were analyzed to determine the ability for PSA to distinguish patients with benign vs adenocarcinoma (Figure 4A). The scatterplot depicts individual variation in PSA levels within each group. There were no significant differences in PSA levels between patients with benign vs adenocarcinoma in the group of patients analyzed in this study (p > 0.05). This is not surprising given that PSA-based assays are known to lack specificity.

As *in silico* findings demonstrated a causal relationship between FLNA and FLNB in PC-3 cells under conditions of hypoxia and lactic acid (Supplementary Figure 2), the relationship between FLNA and FLNB levels was examined in patients with benign cases and in patients with adenocarcinoma. Interestingly, in patients with benign cases, a strong positive correlation between FLNA and FLNB levels was observed (Pearson r = 0.765, p < 0.001; Figure 4B & Supplementary Table 1). In contrast, in patients with adenocarcinoma, the relationship between FLNA and FLNB levels is diminished, as there is no significant correlation between them (Pearson r = 0.030805; p > 0.05; Figure 4B & Supplementary Table 1). It must be noted that three samples were excluded from the analysis in patients with adenocarcinomas, as they were identified as outliers using the robust regression and outlier removal (ROUT) method (Q = 1%). Notably, inclusion of outliers did not affect the results (Pearson r = -0.052; p > 0.05). Additional analysis in patients with adenocarcinoma revealed that PSA levels were positively correlated with Gleason score (Pearson r = 0.7865; p < 0.0001; Supplementary Table 1), and FLNA levels were negatively correlated with Gleason score (Pearson r = -0.3921; p < 0.05; Supplementary Table 1). However, age was not correlated with any variable in patients with benign cases or adenocarcinoma.

## Discussion

This report describes the use of a systems biology approach to identify potential biomarkers for PrCa and provides preliminary validation of this approach. Importantly, a novel finding is that both FLNA and FLNB are secreted and the ability to detect both markers in human biofluids provides a noninvasive means to detect PrCa in patients. In addition, the dysregulation in blood levels of the two markers in patients with adenocarcinoma implicates an important biological link between the filamin proteins in PrCa.

Identification of disease-specific biomarkers requires determining differentials; a process which entails appropriate use of bioinformatics. Here, a Bayesian network structure learning methodology was used to identify novel causal biomarkers of PrCa. While the advantage and theoretical methods for use of Bayesian networks in genomic and proteomic cancer studies have been described [21], few studies have been published that have demonstrated application with biological output using the combined approach presented here.

Clinical utility of molecular markers for disease characterization is dictated by the presence or absence of the markers in easily available biofluids, for example, blood and urine. Proteomic profiling of the secretome is a useful tool for identifying novel markers for diseases. The secretome includes an array of proteins involved in processes such as immune responses and matrix remodeling, which are important mechanisms involved in invasion and metastasis of cancer cells [22,23]. It should be noted that it is possible that the detection of FLNA, FLNB and KRT19 in the secretome of *in vitro* cultures could be the result of release of intracellular proteins from dead cells (necrotic or apoptotic). However, it is unlikely that the levels detected in the present report reflect that released from dead cells. To ensure this, culture conditions were maintained at a confluency that was not overloaded, which allowed for growth and limited death in the brief time period of culture. Second, the lower LOD of MS would be unable to detect the levels of proteins that are released from the few cells that die. Moreover, there is literature that supports that FLNA and FLNB proteins are secreted. FLNA has been detected in mucus from the skin of fish [24] and interestingly, in isolated rat aorta *ex vivo* stimulation with N(omega)-nitro-L-arginine methyl ester-induced secretion of a C-terminal fragment of FLNA [25]. Last, FLNB has been reported to be detected in human plasma [26].

KRT19 is the smallest and atypical in its class of keratins and is altered in cancers including pancreatic, hepatocellular and breast cancer. Altered expression of KRT19 has been demonstrated in bone marrow of metastatic PrCa patients [27]. The absence of secreted KRT19 protein in LNCaP cells compared with that in androgen refractory cell lines suggests utility of KRT19 as a biomarker for differentiating aggressive metastatic forms of PrCa, a concept that is supported by other studies [12,27].

FLNA and FLNB belong to a family of large actin-binding filamins and play a major role in cell migration, vascular development, extracellular signaling and activity of integrins [12]. Reduced expression of FLNC has been observed in prostate tissue of patients [28], and is one of seventeen genes screened in prostate tissue samples in the commercially available Oncotype DX PrCa assay. Prior studies have demonstrated a role for FLNA in normal prostate physiology and in PrCa [13–14,29]. For example, Sun *et al.* demonstrated decreased FLNA intracellular protein expression in prostate tumors that correlated with T stage, lymph node metastasis, clinic stage and Gleason score, but not with age or PSA concentration [14]. Similarly, here FLNA blood levels were found to be negatively associated with Gleason score. However, this study is the first to identify and report FLNB as a potential biomarker for PrCa. Most notably, this report describes that the relationship between FLNA and FLNB is dysregulated in patients with PrCa. It has been proposed that FLNA and FLNB have overlapping roles in cell function [12]. However, the findings presented here suggest a potential nonoverlapping functional link between the two proteins.

It must be noted that variations in intracellular mRNA expression and protein secretion were observed for all three molecules across the PrCa cell lines. Notably, the observation that there was no differential in *FLNA* and *KRT19* mRNA expression after exogenous androgen treatment (R1881) between LnCAP and the androgen-insensitive, DU145 and PC-3 cell lines suggest that transcriptional regulation is independent of androgen sensitivity. Note, we confirmed androgen sensitivity of the cell lines by stimuli-dependent regulation of PSA expression in the cell lines and the regulation of canonical pathways. Moreover, the only stimulus that affected transcriptional regulation of the potential biomarkers was hypoxia in LnCAP and PC-3 cells, which suggests that the regulation of *FLNA*, *FLNB* and *KRT19* by PrCa-relevant stimuli is unlike PSA.

In this report we focused on characterization of the potential biomarkers in PrCa cell lines, but it must be noted that FLNA has been described to be dysregulated in a variety of cancers, including ovarian [30], breast [31] and pancreatic [32]. We note that both FLNA and FLNB were detected in the secretome of pancreatic cell lines, MIA-Paca2 and panc1, and at levels that were not significantly different from PrCa cells (data not shown). Thus, while FLNA and FLNB alone would not be sufficient to rule out other malignancies, inclusion of FLNA and FLNB in a panel (e.g., similar to the Oncotype DX PrCa assay) in conjunction with standard noninvasive PSA-based tests may potentially be used to diagnose PrCa.

The PSA test is the first-line biomarker option to detect PrCa [33], and while beneficial it requires a digital rectal exam and follow-up biopsies to confirm diagnosis. PSA is organ-specific and the processing and activation of pro-PSA to PSA requires the activity of human kallikrein proteins (hk2, hk4 and hk15) and serine proteases encoded by chromosome 19 (in humans), factors that contribute toward lack of specificity in diagnosis of prostatic disease [20]. The main advantage of including additional markers in a blood-based panel along with PSA is the reduction in costs that are associated with invasive follow-up biopsies, these include monetary and patient-related quality of care. Thus, development and inclusion of biomarkers unrelated to PSA is critical. In this report the biomarkers identified appear to be independent of PSA levels *in vitro* and in clinical specimens.

## Conclusion

This report provides evidence to support the use of a novel data-driven strategy to identify causal markers of PrCa. Using a combined *in vitro* and *in silico* approach, FLNB and KRT19 were identified as potential biomarkers for PrCa and FLNA was found to be causally linked to FLNB. These data suggest that the combined use of both markers in a panel may be necessary for detecting PrCa.

## Future perspective

The current method for detection of PrCa includes screening with a PSA blood test followed by a digital rectal exam and prostate biopsy to confirm diagnosis. However, due to the lack of specificity of PSA-based tests and the costs associated with overdiagnosis and unnecessary biopsies there is a critical need for novel noninvasive biomarkers for PrCa that are PSA-independent. The methods described here provide a more rapid approach than traditional methods to identify potential biomarkers of PrCa. In addition, the markers identified here when used in conjunction with other noninvasive blood-based markers in a panel, including PSA, may provide improved predictive power in detecting PrCa. Future efforts should focus on identifying novel biomarkers for PrCa to reduce the burden of overdiagnosis and unnecessary biopsies and associated costs on the patient and healthcare system.

**Table 1.** **Patient demographics.**

**Condition**	**N**	**Mean age (range)**	**Mean PSA (± SEM)**	**Mean number of biopsies**
Benign	14	60.86 (50–69)	6.3 (± 1.3)	9 (± 1.0)
Benign, intraepithelial neoplasia	4	62.3 (57–69)	6.2 (± 0.9)	12.25 (± 3.8)
Benign total	18			
Adenocarcinoma total	28	61.2 (50–69)	15.7 (± 5.0)	10.2 (± 1.1)
By Gleason Score:				
6	7			
7	9			
8	1			
9	2			
Total	46			

PSA: Prostate-specific antigen; SEM: Standard error of the mean.

**Table 2.** **Biomarker ranking in PC-3 cells using Burt’s constraint.**

**Burt's measures**	**Score**			**Rank**		
	**FLNA**	**FLNB**	**KRT19**	**FLNA**	**FLNB**	**KRT19**
Closeness	1.43E-05	1.53E-05	1.57E-05	56	2	1
Constraint	0.500	0.077	0.063	52	2	1
Degree	2.000	13.000	16.000	52	2	1

FLNA: Filamin-A; FLNB: Filamin-B; KRT19: Keratin-19.

**Executive summary**

Proteomic profiling of cancer cells is a promising strategy to identify novel biomarkers for disease.
Secreted proteins theoretically enter bodily fluids and thus represent an easily available matrix for potential biomarkers that can be detected via noninvasive methods.
A novel data-driven, systems biology-based approach was used to identify potential biomarkers for prostate cancer.

**Methods**

The approach used in this study combined *in vitro* perturbation biology and proteomic analysis with *in silico* Bayesian causal analysis and delineation of subnetworks.
The metastatic prostate cancer cell line, PC-3, was exposed to perturbations mimicking the tumor microenvironment (poor oxygenation, low pH and diminished nutrient state) and conditioned media were subjected to proteomic analysis 24 and 48 h after culture.
Proteomic data were analyzed using a Bayesian network inference approach, in which each particular factorization and the choice of parameters are a distinct probabilistic model of the process that created the observed experimental data.
Subnetworks linked to the functional variables were ranked based on their Burt’s constraint score metric, which measures the extent to which nodes (proteins) are connecting to unconnected modules and the relationship redundancy within each of these modules.
Transcriptional regulation and secretion of the identified biomarkers, Filamin-B (FLNB) and Keratin-19 (KRT19), were characterized in in vitro models to validate the findings. Filamin-A (FLNA), a protein causally linked to FLNB, was also included in the analysis to biologically validate the *in vitro* and *in silico* findings.

**Results**

FLNB and KRT19 were identified as very important nodes that were causally influenced by culture conditions of hypoxia and lactic acid in the metastatic prostate cell line, PC-3.
FLNA was found to be causally linked to FLNB.
The mRNA expression levels of *FLNA*, *FLNB* and *KRT19* were differentially expressed in a panel of prostate cancer cell lines (LnCAP, DU145 and PC-3) compared to normal human prostate epithelial cells.
Exposure to prostate cancer-relevant stimuli differentially affects transcriptional regulation of the biomarkers in the panel of cell lines and their regulation appears to be independent of prostate-specific antigen expression.
Both FLNA and FLNB were found in the secretome of each cancer cell line examined. KRT19 was detected in DU145 and PC-3, but not in LnCAP cell lines.
Both FLNA and FLNB were detected in plasma from men with suspected prostate cancer.
In patients with benign cases a strong positive correlation between blood levels of the two markers was observed (Pearson r = 0.765; p < 0.001).
In patients with adenocarcinoma the relationship between FLNA and FLNB blood levels was diminished (Pearson r = 0.030805; p > 0.05).

**Discussion**

The use of the secretome provides a surrogate to identify potentially noninvasive markers.
The secretome includes an array of proteins involved in many processes, including mechanisms important for invasion and metastasis of cancer cells.
Prior studies have demonstrated a role for FLNA in normal prostate physiology and in prostate cancer.
This report is the first to identify FLNB as a potential biomarker for prostate cancer.
The *in vitro* and *in silico* findings demonstrate that FLNB is causally associated with FLNA in prostate cancer.
Clinical data provide preliminary support of a relationship between FLNB and FLNA that is dysregulated in patients with prostate cancer.

**Conclusion**

The findings demonstrate the use of a systems biology approach to identify potential biomarkers for prostate cancer and provide preliminary validation of this approach.
The combined use of both markers in a panel in conjunction with prostate-specific antigen may potentially yield better predictive power for detection of prostate cancer than prostate-specific antigen alone.
