# Supplementary figures and images for: Identification of Filamin-A and -B as potential biomarkers for prostate cancer
Source: Future Sci OA. 2016 Dec 22;3(1):FSO161. doi: 10.4155/fsoa-2016-0065 (PMC5351499; doi:10.4155/fsoa-2016-0065)

Supplemental Figure 1

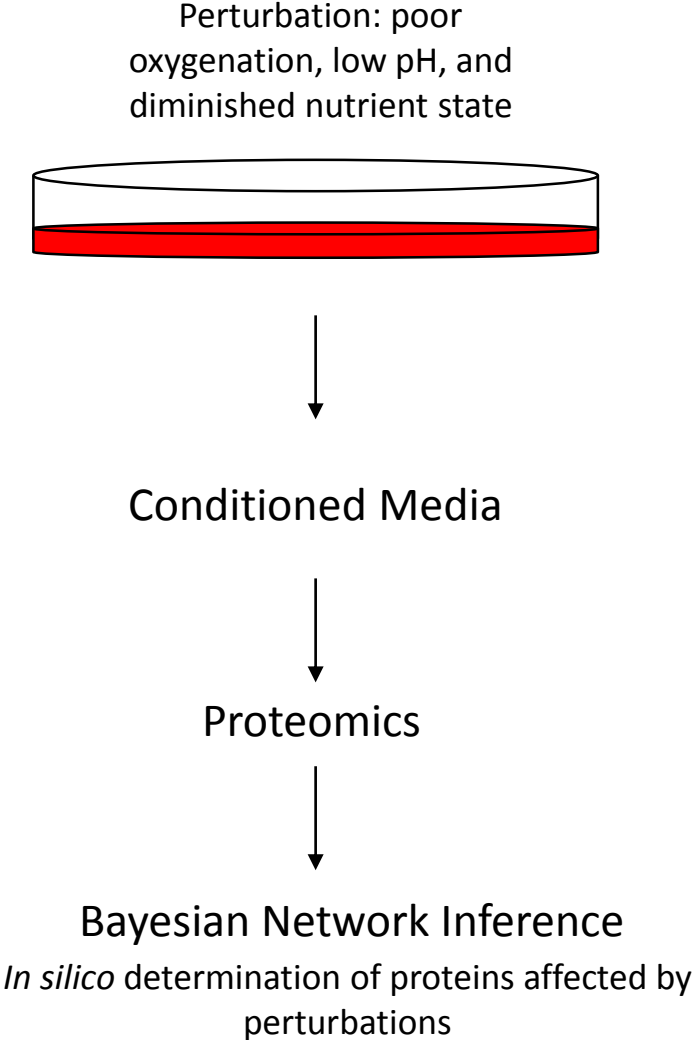

Supplement: Supplementary file 1 [file fsoa-03-161-s1.pdf]

Supplemental Figure 2

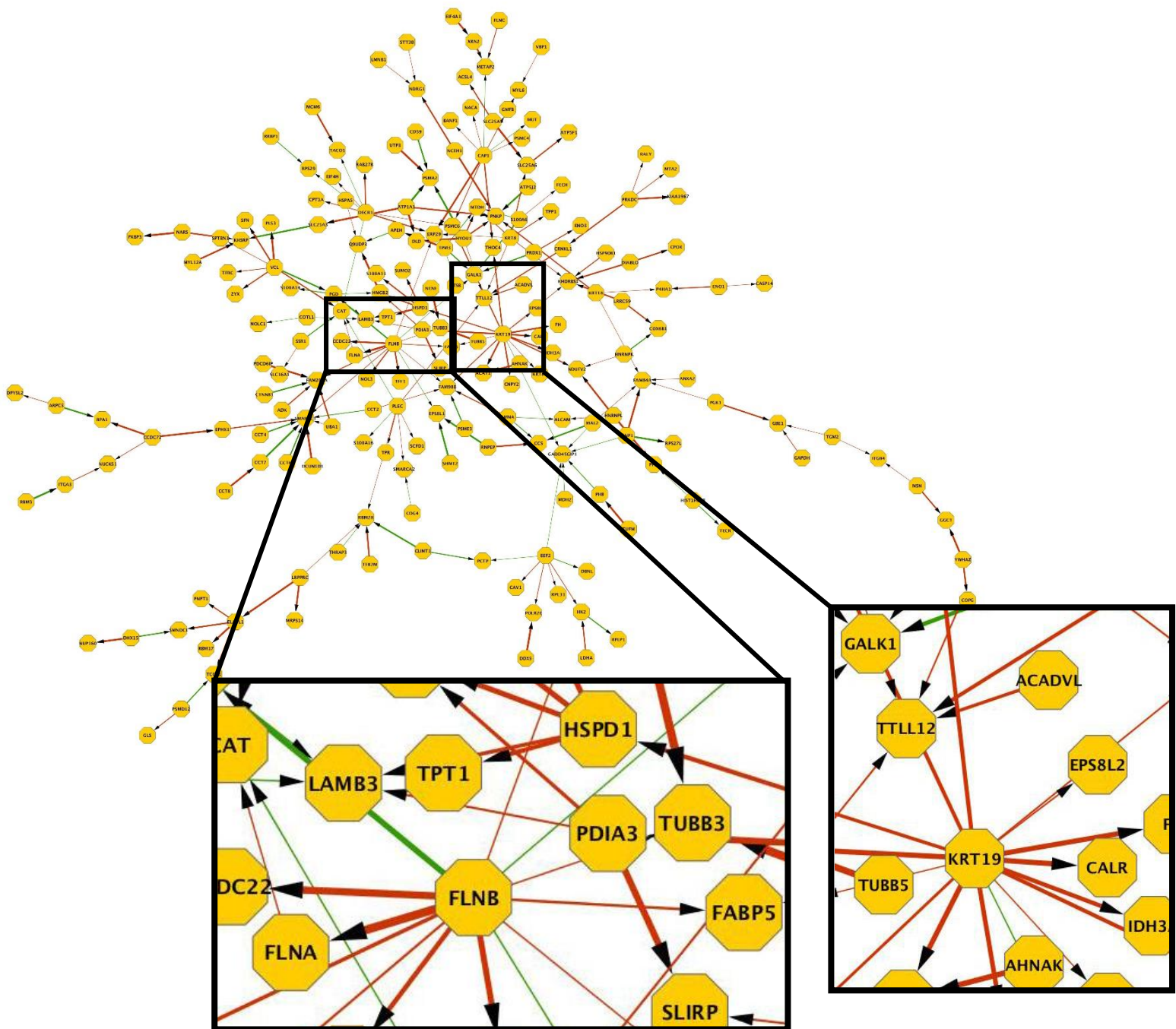

Supplement: Supplementary file 2 [file fsoa-03-161-s2.pdf]

# Supplemental Figure 3

A

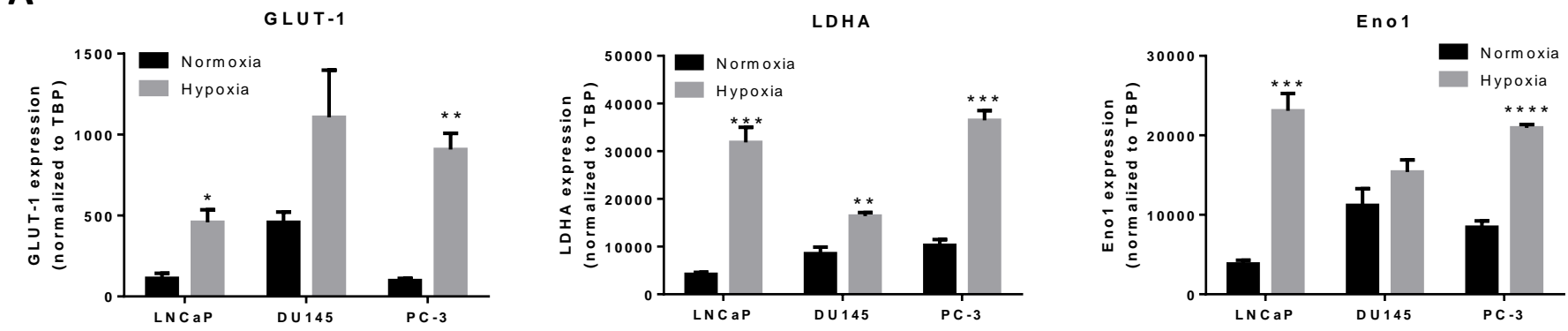

B

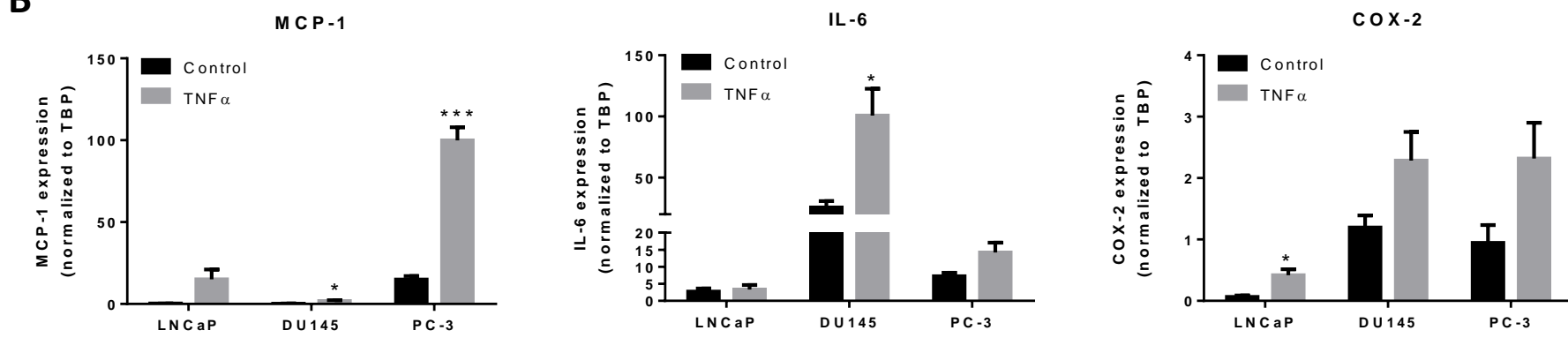

C

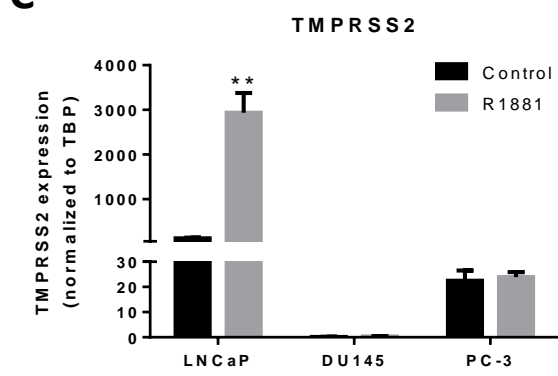

Supplement: Supplementary file 3 [file fsoa-03-161-s3.pdf]
