# Supplementary material for: Identification of Filamin-A and -B as potential biomarkers for prostate cancer
Source: Future Sci OA. 2016 Dec 22;3(1):FSO161. doi: 10.4155/fsoa-2016-0065 (PMC5351499; doi:10.4155/fsoa-2016-0065)
Supplement: Supplementary file 4 [file fsoa-03-161-s4.docx]

**Supplemental Table S1.**

| **Pearson r in Benign** | | | | | | **Corresponding p - values** | | | | | |
| --- | --- | --- | --- | --- | --- | --- | --- | --- | --- | --- | --- |
|  | **PSA** | **FLNA** | **FLNB** | **Age** |  |  | **PSA** | **FLNA** | **FLNB** | **Age** |  |
| **PSA** |  | -0.157 | -0.178 | -0.331 |  | **PSA** |  | 0.535 | 0.495 | 0.179 |  |
| **FLNA** | -0.157 |  | 0.765 | 0.372 |  | **FLNA** | 0.535 |  | 0.000 | 0.129 |  |
| **FLNB** | -0.178 | 0.765 |  | 0.334 |  | **FLNB** | 0.495 | 0.000 |  | 0.190 |  |
| **Age** | -0.331 | 0.372 | 0.334 |  |  | **Age** | 0.179 | 0.129 | 0.190 |  |  |
| **Pearson r in Adenocarcinoma** | | | | | | **Corresponding p - values** | | | | | |
|  | **PSA** | **FLNA** | **FLNB** | **Age** | **Gleason score** |  | **PSA** | **FLNA** | **FLNB** | **Age** | **Gleason score** |
| **PSA** |  | 0.161 | -0.085 | -0.096 | 0.786 | **PSA** |  | 0.412 | 0.694 | 0.627 | 0.000 |
| **FLNA** | 0.161 |  | -0.052 | 0.040 | -0.392 | **FLNA** | 0.412 |  | 0.810 | 0.839 | 0.048 |
| **FLNB** | -0.085 | -0.052 |  | -0.015 | -0.155 | **FLNB** | 0.694 | 0.810 |  | 0.943 | 0.479 |
| **Age** | -0.096 | 0.040 | -0.015 |  | -0.151 | **Age** | 0.627 | 0.839 | 0.943 |  | 0.462 |
| **Gleason score** | 0.786 | -0.392 | -0.155 | -0.151 |  | **Gleason score** | 0.000 | 0.048 | 0.479 | 0.462 |  |
